# Comprehensive Virome Profiling of Apple Mosaic Disease-Affected Trees in Iran Using RT-PCR and Next-Generation Sequencing

**DOI:** 10.3390/v17070979

**Published:** 2025-07-13

**Authors:** Anahita Hamedi, Farshad Rakhshandehroo, Mohammad Reza Safarnejad, Gholamreza Salehi Jouzani, Amani Ben Slimen, Toufic Elbeaino

**Affiliations:** 1 Department of Plant Protection, SR. C., Islamic Azad University, Tehran 14515-775, Iran; anahita_hamedi@yahoo.com; 2 Department of Plant Viruses, Iranian Research Institute of Plant Protection, Agricultural Research, Education and Extension Organization (AREEO), Tehran 19395-1454, Iran; mrsafarnejad@yahoo.com; 3 Microbial Biotechnology Department, Agricultural Biotechnology Research Institute of Iran (ABRII), Agricultural Research, Education and Extension Organization (AREEO), SPII Campus, Karaj 31535-1897, Iran; gsalehi@abrii.ac.ir; 4 International Centre for Advanced Mediterranean Agronomic Studies (CIHEAM Bari), 70010 Valenzano, Italy; b.slimen@iamb.it; 5 National Research Council of Italy (CNR), Institute for Sustainable Plant Protection (IPSP), Piazzale Enrico Fermi, 80055 Portici, Italy

**Keywords:** viruses, viroids, detection, molecular characterization, phylogenetic analysis

## Abstract

Apples (*Malus domestica*), one of Iran’s oldest cultivated fruit crops, hold considerable economic importance. In this study, 170 apple leaf samples representing various commercial cultivars were collected across the country. RT-PCR screening targeted five common apple-infecting viruses and two viroids: apple chlorotic leaf spot virus (ACLSV), apple stem pitting virus (ASPV), apple stem grooving virus (ASGV), apple green crinkle-associated virus (AGCaV), apple mosaic virus (ApMV), apple scar skin viroid (ASSVd), and hop stunt viroid (HSVd). To identify additional or novel agents, 40 RT-PCR-negative samples were pooled into two composite groups and analyzed using next-generation sequencing (NGS). NGS was also performed on individual samples with mixed infections to retrieve full genomes. RT-PCR confirmed the presence of ACLSV, ASPV, ASGV, AGCaV, ApMV, and HSVd. NGS further revealed three additional pathogens: citrus concave gum-associated virus (CCGaV), apple hammerhead viroid (AHVd), and apricot vein clearing-associated virus (AVCaV), which were subsequently detected across the collection by RT-PCR. AGCaV was most prevalent (47.6%), followed by ACLSV (45.8%), HSVd (27.6%), AVCaV (20.5%), ASGV (17%), AHVd (15.2%), ASPV (14.1%), CCGaV (4.7%), and ApMV (3.5%). Mixed infections occurred in 67% of samples. Phylogenetic analysis based on CP genes (ACLSV, ASGV, AGCaV) and full genomes (AVCaV, AHVd) clustered Iranian isolates together, suggesting a common origin. This is the first report in Iran of AGCaV, CCGaV, ApMV, and AVCaV in apple, and notably, the first global report of AVCaV in a non-Prunus host. The findings provide the first comprehensive assessment of the sanitary status of apple trees in Iran.

## 1. Introduction

Apple (*Malus × domestica* Borkh.), a member of the Rosaceae family, is one of the most widely cultivated fruit crops worldwide. Iran ranks sixth in global apple production [1], with major cultivation areas located in West Azerbaijan, East Azerbaijan, Tehran, Isfahan, Khorasan Razavi, and Ardabil provinces. Apple is predominantly propagated vegetatively through grafting, cuttings, or micropropagation, which facilitates the transmission of viruses, many of which remain latent and symptomless in commercial cultivars [2]. Due to the difficulty of managing viral infections in apple, the use of certified, virus-free propagation material remains the most effective strategy to prevent disease spread [3,4].

Among the most widespread and economically damaging viral and viroidal pathogens of apples are apple chlorotic leaf spot virus (ACLSV; genus *Trichovirus*), apple stem grooving virus (ASGV), apple green crinkle-associated virus (AGCaV; both genus *Foveavirus*), apple stem pitting virus (ASPV; genus *Capillovirus*), apple mosaic virus (ApMV; genus *Ilarvirus*), and apple scar skin viroid (ASSVd, genus *Apscaviroid*) [5,6]. These pathogens are often found in mixed infections. While some viruses, such as ACLSV, ASGV, and ASPV, are asymptomatic in many commercial cultivars, susceptible varieties may exhibit symptoms including leaf deformation, stem grooving, poor graft union development, and reduced fruit yield and quality [7]. Given their potential economic impact, these viruses have been listed on the EPPO Alert List [8].

Traditional diagnostic approaches, including symptom observation, ELISA, RT-qPCR, RPA, and LAMP, are commonly used to detect these pathogens [9]. However, their accuracy can be limited by the genetic variability of viral populations, which may result in false negatives due to mismatches in antibody recognition or primer binding [10]. Additionally, these techniques can be labor-intensive and inefficient for next-generation screening (NGS). NGS addresses these limitations by enabling the comprehensive, unbiased detection of both known and novel viruses and viroids, including those present at low titers and in asymptomatic plants, without requiring prior genomic information [11,12]. HTS has proven effective in uncovering complex viromes in symptomatic and asymptomatic apple trees [2,13,14,15]. In Iran, previous studies have reported the presence of ACLSV, ASGV, ASPV, tomato ringspot virus (ToRSV), HSVd, and AHVd in diseased apple cultivars, primarily using ELISA and RT-PCR methods [16,17,18,19,20,21]. Among these, ACLSV and ASPV were the most prevalent, occurring in both symptomatic and asymptomatic trees. In addition, these studies highlighted regional differences and the genetic diversity of virus isolates across Iranian apple-growing regions [18]. Although ASGV, ASPV, and ACLSV are generally associated with latent infections in most apple cultivars [6,21], they have also been linked to symptoms such as stem grooving, leaf malformation, and epinasty in susceptible varieties [8,21]. In the Alborz province of Iran, the presence of these viruses in local apple cultivars has been previously confirmed using ELISA and RT-PCR analyses on samples collected from symptomatic trees [17,19]. However, the overall incidence and genetic diversity of major viruses associated with Apple Mosaic Disease (AMD) in Iranian apple orchards have not been comprehensively investigated.

In this study, we used RT-PCR to further examine the distribution of viruses and viroids previously revealed by HTS across different commercially grown apple cultivars. Additionally, we report for the first time in Iran the complete genome sequences of ACLSV, ASPV, ASGV, AGCaV, ApMV, AVCaV, CCGaV, AHVd, and HSVd identified in apple trees. While near full-length sequences of two ASGV isolates from Iran had been reported earlier [21], our findings provide the first full-length genome data for several of these viruses. Notably, the complete genome sequences of AVCaV detected in multiple apple cultivars offer new insights into the molecular diversity and potential host range expansion of this recently identified virus. Despite these findings, no comprehensive virome analysis has been performed on commercially cultivated apple trees in Iran. The present study aimed to fill this gap by investigating the virome associated with mosaic and malformation symptoms in apple orchards across major production areas. Specific RT-PCR assays were first used to detect key viruses and viroids, and subsequently, NGS was performed on pooled samples that resulted in RT-PCR-negative results for the pathogens herein investigated. Several known and previously unreported viruses and viroids were identified, providing new insights into the diversity of apple-infecting pathogens in Iran, for which their results are hereafter reported and discussed.

## 2. Materials and Methods

### 2.1. Plant Material and Sample Preparation

**Figure 1:**
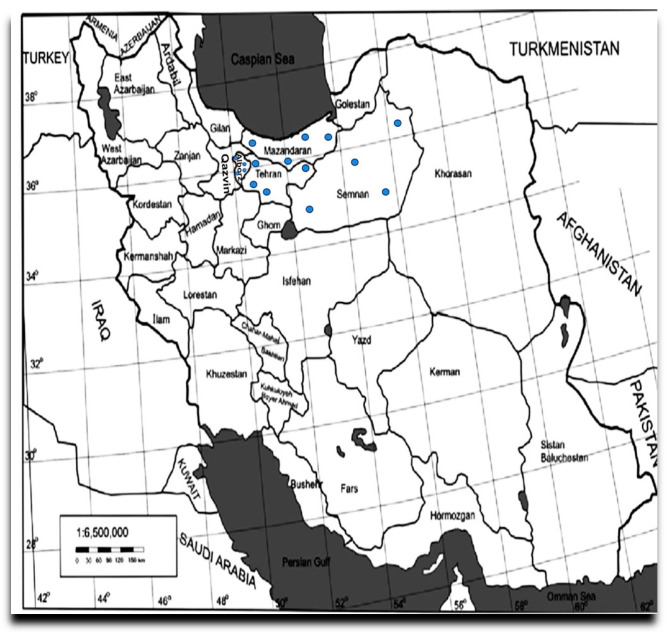
Map showing the locations of sampling sites located in four provinces of Iran. The samples were collected from the Central and Northern regions in Iran at the 20 locations indicated by dots on the map.

**Figure 2:**
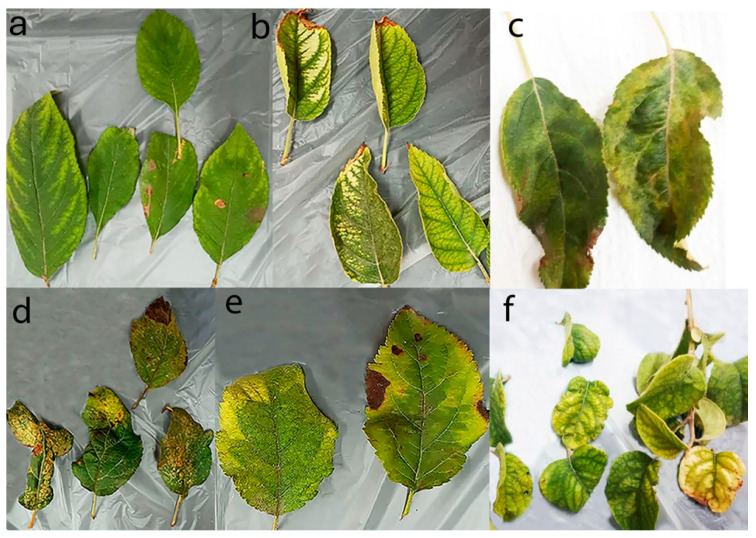
Apple leaves showing symptoms of interveinal chlorosis (**a**); vein banding (**b**); mosaic (**c**); deformation with necrotic ring spot on cultivar Golden Delicious (**d**); mosaic with necrotic leaf spots on cultivar Red Delicious (**e**); mosaic, deformation and yellowing on samples subjected to NGS (**f**).

Leaf samples exhibiting typical AMD symptoms, including leaf deformation, chlorotic or necrotic spots, and yellowing of veins, were collected from 170 apple trees of various cultivars during the 2020 and 2021 growing seasons across 20 orchards located in four major apple-producing provinces of Iran: Alborz, Mazandaran, Semnan, and Tehran (Figure 1). However, the trees exhibiting virus-like symptoms were prioritized during sampling (Figure 2).

Many of the surveyed orchards were 7–10 years old and typically included more than one cultivar. Most of the samples were collected from the Gala, Golden Delicious, and Red Delicious cultivars, as they are the most widely planted in the surveyed regions, and many of their trees exhibited severe symptoms. Additional samples were taken from other major cultivars, including Granny Smith, Starking, and the local variety Shafi Abadi. Each sample consisted of 5 leaves collected from 2 to 3 different branches of a single tree. The collected samples were placed in plastic bags and immediately transported to the laboratory. For each sample, the sampling region, time, and observed symptoms were recorded. Subsequently, the samples were flash-frozen in liquid nitrogen and stored at –80 °C for further analysis.

The total nucleic acids (TNA) were extracted from fresh leaves according to Foissac et al. [22]. In brief, 200 mg of tissue from leaf veins was homogenized in 1 mL grinding buffer (4.0 M guanidine thiocyanate, 0.2 M NaOAc pH 5.2, 25 mM EDTA, 1.0 M KOAc pH 5.0, and 2.5% *w*/*v* PVP-40). After centrifugation for 10 min at 13,000× *g*, the supernatant was mixed with 6 M sodium iodide and 0.15 M sodium sulfite, 150 µL ethanol, and 40 µL silica particles in suspension (1 g/mL, pH 2.0). After stripping by heat treatment in sterile water (70 °C for 3 min) and centrifugation for 3 min at 16,000× *g*, recovered TNAs were quantified using Nanodrop ( Thermo Scientific, Waltham, MA, USA) at wavelengths of 230, 260, and 280 nm. and stored at −20 °C until use.

### 2.2. RT-PCR for Viruses and Viroids Detection

RT-PCR assays were conducted on individual leaf samples using virus- and viroid-specific primers (Supplementary Table S1) targeting conserved regions of the coat protein gene (for viruses) or genome (for viroids) to detect seven common apple pathogens: ACLSV, ASPV, ASGV, AGCaV, ApMV, ASSVd, and HSVd. In addition, RT-PCR assays were performed to ascertain the presence of CCGaV), AVCaV, AHVd, whose presence was revealed by NGS. Accordingly, the relative incidence of each pathogen in the collected samples was also assessed.

The TNAs were reverse-transcribed using 0.5 µg of a random hexanucleotide primer mixture (Roche Diagnostics, Mannheim, Germany) and 200 units of Moloney murine leukemia virus (M-MLV) reverse transcriptase (Invitrogen, Waltham, MA, USA) in a 20 µL reaction, incubated for 1 h at 39 °C according to the manufacturer’s protocol. RT-PCR conditions for detecting CCGaV, HSVd, AHVd, and ASSVd, followed the protocols described in the references listed in Supplementary Table S1. For the detection of ACLSV, AGCaV, ApMV, ASGV, ASPV, and AVCaV (primers designed in this study), RT-PCR was performed using 2 µL of cDNA in a µL reaction mixture containing 2 µL of 10× Taq buffer (Promega, Madison, WI, USA), 1.5 mM MgCl_2_, 0 µL of 10 mM dNTPs, 0 µL each of 10 μM forward and reverse primers, and 0 µL of Taq DNA polymerase (5 U/μL). Cycling consisted of an initial denaturation at 94 °C for 5 min, followed by 39 cycles of denaturation at 94 °C for 30 s, annealing at 52 °C for 30 s, and extension at 72 °C for 40 s, with a final extension at 72 °C for 5 min.

Amplified PCR products were separated by electrophoresis on a 1.5% agarose gel in 1× TAE buffer, stained with GelRed 10,000X (Biotium, Hayward, CA, USA), and visualized under UV light. For Sanger sequencing, target amplicons were generated using DreamTaq DNA polymerase (Thermo Fisher Scientific, Milan, Italy), excised from the gel, and purified with the GeneJET Gel Extraction Kit (Thermo Fisher Scientific, Waltham, Massachusetts, USA). Purified products were either directly sequenced or cloned using CloneJET PCR cloning Kit (Thermo Fisher) and transformed into DH5α competent cells. Positive clones were identified by blue-white screening, colony PCR, and restriction digestion. Confirmed clones of selected viruses and viroids were sequenced bidirectionally by Eurofins Genomics (Ebersberg, Germany), and the resulting sequences were submitted to NCBI GenBank (Table 1).

### 2.3. Next-Generation Sequencing (NGS)

NGS was conducted on two composite TNA samples, designated A1 and A2. Each composite represented pooled material from 20 individual apple trees that tested negative for the targeted viruses and viroids in this study. Sample A1 comprised trees collected from Semnan and Tehran provinces (central and eastern Iran), whereas sample A2 included trees from Alborz and Mazandaran provinces (northern Iran). In addition, NGS was performed on four individual apple tree samples that exhibited distinct combinations of viral and viroid infections as determined by RT-PCR, with the aim of obtaining complete genome sequences of all identified pathogens.

For the A1 and A2 libraries, 1 µg of total RNA was prepared per library by pooling 50 ng of RNA from each of the 20 leaf samples, totaling 40 individual trees across both composite samples. This pooling strategy reduced the number of samples submitted for sequencing while ensuring broad representation in terms of cultivar and possible infection. It also increased the likelihood of detecting both known apple-infecting viruses not targeted by the RT-PCR assays, as well as potentially novel viral agents present in Iranian apple orchards. For the individual plant libraries, 200 ng of RNA from each of the four selected apple trees was used, each harboring a unique combination of viral and viroid infections.

### 2.4. Bioinformatic Analysis

Bioinformatic analysis of the raw NGS data was conducted using CLC Genomics Workbench v10.1.1 (Qiagen Bioinformatics, Hilden, Germany) and Geneious Prime v2020.2.5 (Biomatters Ltd., Auckland, New Zealand). Raw sequencing reads were first filtered to remove adapter sequences and low-quality reads, then mapped to the *Malus domestica* reference genome (accession number GCA_002114115.1) to eliminate host-derived sequences [23]. Unmapped reads were subsequently de novo assembled into contigs, and those longer than 200 nucleotides were annotated via BLASTN/X analysis using a cutoff e-value of 10^−4^ against local and online virus, viroid, and nt/nr databases (https://www.ncbi.nlm.nih.gov/orffinder, accessed on 10 November 2024).

To recover full or near-complete viral and viroid genomes, contigs showing virus/viroid similarity were extended by iterative read mapping using Geneious Prime. Contigs with 70–85% identity to known viral sequences were further analyzed by additional BLAST searches against the NCBI database to ensure that potentially novel viruses were not overlooked. The abundance of each detected virus was estimated by mapping reads to the corresponding reference viral genome. Assembled viral genomes with over 90% query coverage compared with their reference sequences were selected for further analysis. Final nucleotide sequences obtained from Sanger Sequencing and through NGS were deposited in GenBank and aligned with reference sequences of the corresponding viruses and viroids, as well as with the most similar homologous sequences available in GenBank, using the BLASTN tool provided by NCBI (https://blast.ncbi.nlm.nih.gov/Blast.cgi, accessed on 30 September 2024).

### 2.5. Phylogenetic Analysis

Phylogenetic trees were constructed based on nucleotide sequence alignments using the “Maximum Likelihood” (ML) method of the Kimura two-parameter model, with 1000 bootstrap replicates, implemented in MEGA version 11.0.10 [24]. The analyses regarded the complete CP gene sequences of AGCaV, ASGV, and ASPV as being among those viruses that showed high incidence across various apple cultivars in the surveyed regions. For coat protein (CP)-based phylogenetic analysis, reference isolates were selected from GenBank to represent a broad geographic distribution, ensuring inclusion of at least one representative from each major continent. In addition, complete genome sequences of AVCaV and AHVd obtained in this study were analyzed alongside corresponding full-length sequences available in GenBank to assess their phylogenetic relationships.

## 3. Results

### 3.1. Detection and Prevalence of Apple Viruses and Viroids Using RT-PCR Assays

RT-PCR analysis revealed the presence of AGCaV, ACLSV, ASPV, ASGV, ApMV, and HSVd in apple samples at varying infection levels, while ASCVd was not detected. Overall, 81 out of 170 samples (47.6%) tested positive for at least one virus (Table 2). AGCaV was the most prevalent (47.6%), followed by ACLSV (45.8%), HSVd (27.6%), ASGV (17%), ASPV (14.1%), and ApMV (3.5%). AGCaV was detected in all surveyed provinces, with the highest incidence in Mazandaran (69.6%), followed by Semnan (50%), Alborz (28.1%), and Tehran (23.3%). ACLSV was also widespread, with incidence rates of 57.1% (Mazandaran), 21.1% (Semnan), 65.6% (Alborz), and 46.6% (Tehran). Notably, Alborz province showed the highest infection rates for ACLSV (65.6%) and ApMV (6.2%), whereas Mazandaran had the highest rate for AGCaV (69.6%). The highest incidences of ASGV (23%) and ASPV (15.3%) were recorded in Semnan province (Table 2).

### 3.2. NGS-Identified Viruses and Viroids in Apple and Their Prevalence Validated by RT-PCR

NGS was conducted on total RNA extracted from leaves of virus- and viroid-free apple cultivars, using two composite samples (A1 and A2). Illumina NovaSeq 6000 sequencing platform generated 36,652,324 and 43,845,194 paired-end reads for A1 and A2, respectively, after quality filtering and adapter trimming (Supplementary Table S2). Following host genome subtraction, 5,315,765 reads (A1) and 3,025,604 reads (A2) were subjected to de novo assembly, and the resulting contigs were screened in BLASTn and BLAST X for viruses and viroids search. Libraries analysis of NGS-A1 and -A2 revealed the presence of two viruses and one viroid that initially were not contemplated in our virome screening, i.e., apple hammerhead viroid (AHVd; genus *Pelamoviroid*, family *Avsunviroidae*), citrus concave gum-associated virus (CCGaV; genus *Coguvirus*, family *Phenuiviridae*), and, surprisingly, apricot vein clearing-associated virus (AVCaV; genus *Prunevirus*), which has never been reported before in the literature to infect non-Prunus hosts. Comparative analysis revealed that sample A2, composed of apple trees from Mazandaran and Alborz provinces, showed higher levels of virus- and viroid-associated reads and a greater number of contigs compared with sample A1 from Semnan and Tehran, indicating a higher viral load and diversity in A2 (Supplementary Table S2). To assess the incidence and distribution of the three detected pathogens, all 170 apple samples were analyzed by RT-PCR using specific primers across different cultivars and provinces. The results show that AHVd was detected in 15.2% of the samples, while AVCaV and CCGaV were present in 20.5% and 4.7% of the samples, respectively (Table 2). In addition, the highest incidence of AVCaV was recorded in Alborz province, with an infection rate of 46.8%, followed by AHVd at 25% in the same region. In contrast, CCGaV was predominantly detected in Mazandaran province (Table 2).

Combining the deep sequencing technique and RT-PCR data revealed that most apple trees (67%) were infected with multiple viruses and/or viroids, with up to six pathogens detected in a single tree (Table 2). Among the eight viruses and two viroids tested, the most common mixed infection was AGCaV+AVCaV+ASGV+HSVd, present in 47% of infected trees, followed by AVCaV+AGCaV+ASGV+ACLSV+ApMV+HSVd (33.5%) and AVCaV+AGCaV+ASGV+ApMV+HSVd+AHVd (21.7%). A rare triple infection involving CCGaV, AVCaV, and AHVd was also detected in 11% of the cases.

### 3.3. Regional and Varietal Distribution of Virus and Viroid Infections

Regarding regional distribution, both HSVd and AHVd were detected in all surveyed regions, with higher incidence in the central part of Iran (42.6%) compared with the northern and eastern regions (23.1%). Mixed infections of HSVd and AHVd were observed in 10.5% of the tested samples, while some samples were negative for both viroids. Disease symptoms were observed in all surveyed cultivars across all regions, though the severity and incidence varied. High infection rates of AGCaV and ACLSV were recorded, identifying them as the most prevalent viruses among the tested cultivars (Table 3). Among cultivars, Red Delicious, Golden Delicious, and the local variety Shafi Abadi exhibited the highest infection rates for most of the studied viruses (Table 3). The Gala cultivar showed a comparatively lower infection rate, with only ASPV (25%), ACLSV (6.4%), AGCaV (4.9%), AHVd (19.2%), and HSVd (6.3%) detected. No infections with ASGV, AVCaV, ApMV, or CCGaV were found in Gala trees, suggesting a potential tolerance of this cultivar to multiple viral infections.

In Granny Smith and Starking cultivars, ASPV and ASGV were detected at low frequencies, 8.3% and 3.4% in Granny Smith, and 4.1% and 6.5% in Starking, respectively, mainly in Semnan and Mazandaran provinces. AVCaV, ApMV, and CCGaV were not detected in these cultivars across the surveyed regions. HSVd and AHVd were consistently present in Granny Smith (8.5% and 11.5%) and Starking (6.8% and 7.6%), although AHVd was absent in these cultivars in Semnan province (Table 3).

Symptoms such as necrotic leaf spots, chlorotic mottling, and leaf deformation were frequently observed in HSVd- and AHVd-infected trees across all cultivars and locations. However, establishing a definitive correlation between specific symptoms and individual pathogens is a major challenge in plant virology. Such analyses require controlled studies using plant material infected with single pathogens under defined conditions, material that is rarely available, especially given the high frequency of mixed infections in field-grown trees.

### 3.4. Full Genome Sequencing of Detected Viruses and Viroids

NGS was carried out on total RNA extracted from four individual apple trees infected with multiple viruses and viroids to obtain full-length genome sequences of the pathogens previously identified through RT-PCR and NGS. This allowed the generation of high-quality full reference genomes that can support future diagnostic viral and viroidal analysis development based on virus/viroid-specific isolates.

The NGS data generated from A1 and A2 composite samples (Supplementary Table S2) demonstrate the effectiveness of Illumina NovaSeq 6000 in generating high-quality sequencing data, with over 36 million reads per sample and a high proportion mapping to the *Malus domestica* genome. Despite the dominance of host reads, the platform successfully detected viral and viroidal sequences, with Sample A2 showing particularly notable proportions (0.51% viral and 0.24% viroidal). These results highlight the platform’s sensitivity and suitability for identifying low-abundance pathogens in plant samples. High-quality reads and de novo assembled contigs enabled the reconstruction of near-complete pathogen genomes with 96.3% to 98.6% coverage. Overlapping regions between NGS-derived partial sequences and RT-PCR amplicons allowed for the complete assembly of viral and viroid genomes. These sequences were deposited in GenBank under accession numbers OR537850 to OR537861 (Table 1).

Briefly, the ACLSV genome (Damavand-A2, 7,555 nt; OR537850) showed 88% identity with French isolate p863 and 79.5% with Canadian isolate 13C258. AGCaV (Alborz-A5, 9266 nt; OR537851) shared 90% identity with Aurora-1 (Canada) and 84.4% with CYD (Italy). AVCaV (Damavand-A5, 8,358 nt; OR537852) was similar in size and organization to U.S. and French isolates, with conserved ORF structure. ASPV (Damavand-A6, 9258 nt; OR537853) showed 86% identity with isolate 13TF189A and 78% with PO13357 (France). ASGV (Tehran-A4, 6,486 nt; OR537854) encoded two ORFs, with a nested movement protein gene. CCGaV (Semnan-A10; RNA1: 6658 nt, RNA2: 2706 nt; MZ926713, MZ926714) had 99% identity with the Chinese apple isolate Weihai. ApMV (Alborz-A2; RNA1: 3440 nt, RNA2: 2979 nt, RNA3: 2056 nt; OR537857–OR537859) showed 89–90% identity with Indian isolates.

Viroid assemblies revealed complete circular genomes for AHVd (435 nt; Tehran-A5, OR537860) and HSVd (300 nt; Mazandaran-A7, OR537861), confirmed by RT-PCR using sequence-specific primers. AVCaV, ASPV, ApMV RNA1, and CCGaV RNA1 showed >99% query coverage with reference genomes; ACLSV, ASGV, AGCaV, ApMV RNA2/3, and CCGaV RNA2 showed 95–98%.

### 3.5. Phylogenetic and Sequence Variability Analyses

As AGCaV, ACLSV, and ASGV were the most frequently detected viruses by RT-PCR, their molecular and phylogenetic analyses were investigated based on the CP gene sequences. Complete CP gene sequences of three Iranian ACLSV and three ASGV isolates were cloned and deposited in GenBank (Acc. Nos. OR537862–OR537867; Table 4). For AGCaV, partial CP sequences (630 nt) were submitted under Acc. Nos. OR570871–OR570873. BLASTN analysis revealed nt identities of 99%, 84–88%, and 84–92%, and aa identities of 100%, 90–94%, and 85–94%, for ASGV, ACLSV, and AGCaV, respectively, relative to reference isolates. The lowest sequence identities for ACLSV and AGCaV were observed between isolates from the USA and Brazil, with nt/aa identities of 80%/84% and 82%/85%, respectively. The most divergent ASGV isolate was Nagami (Japan), with 90% nt and 95% aa identity to reference sequences. Overall, ACLSV and AGCaV showed greater sequence variability than ASGV.

The complete genome of the Iranian AVCaV isolate Damavand-A5 (Acc. No. OR537852) showed 100% nt and aa identity with the American (PC01, MK170168) and French (Pair, KM507062) isolates from *Prunus mume* and *P. salicina*, respectively. In contrast, it shared only 84% nt and 89% aa identity with the Italian isolate VC (HG008921) from *P. armeniaca*, and 94% nt and 95% aa identity with the Iranian almond isolate Sh20 (ON169997) (Table 4). To confirm RT-PCR detections, representative CP sequences from ApMV, ASPV, AVCaV, CCGaV, and HSVd were sequenced and deposited under Acc. Nos. OR570869–OR570870, OR537868, OR591481, and OR570874, respectively. BLASTN analyses showed 86–96%, 85–97%, 99–100%, and 92–100% nt identity for ApMV, ASPV, AVCaV, and CCGaV, respectively. HSVd sequences showed 95–99% identity with known GenBank sequences.

**Figure 3:**
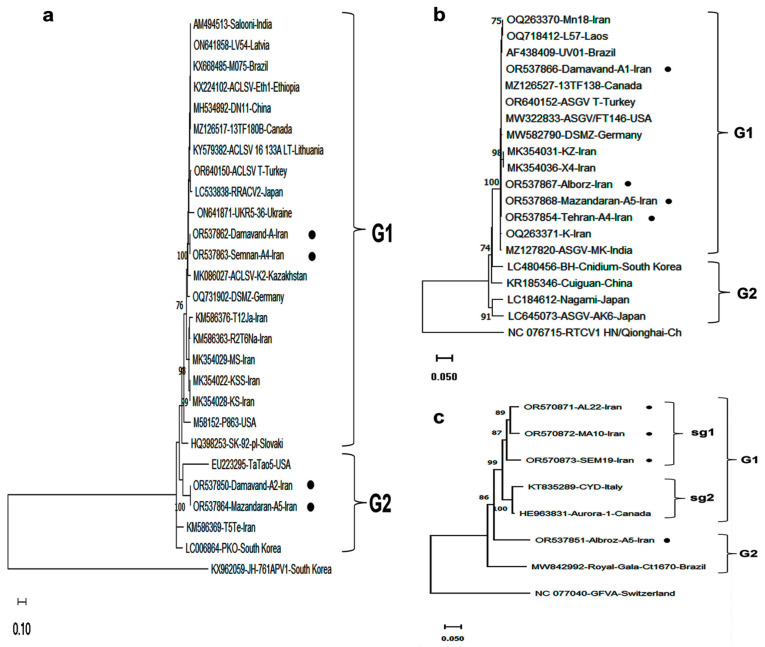
Phylogenetic tree based on the complete CP gene nucleotide sequences of 3 Iranian isolates: (**a**) ACLSV, (**b**) ASGV, and (**c**) AGCaV, together with homologous sequences retrieved from the NCBI database. Isolates are labeled with accession number/isolate name/country. Trees were constructed using MEGA 11.0 with the maximum likelihood (ML) method and the Kimura two-parameter model, with 1000 bootstrap replicates. Bootstrap values >70% are shown. Scale bars represent the number of nucleotide substitutions per site. Iranian isolates detected in this research are marked with black dots. Outgroup sequences used were *Asian prunus virus 1* (KX962059) for ACLSV, *Rubber tree capillovirus 1* (NC_076715) for ASGV, and *Grapevine foveavirus A* (NC_077040) for AGCaV. See Table 1 and Table 4 for accession details.

Phylogenetic trees based on CP gene sequences of ACLSV, ASGV, and AGCaV grouped isolates into two major clades (G1 and G2) (Figure 3). Iranian ACLSV isolates formed two subgroups within G1, clustering with both local and international isolates. ASGV isolates from Iran were restricted to G1, while those from East Asia (China, Japan, South Korea) grouped in G2. AGCaV sequences showed broader distribution, with Iranian isolates found in both subgroups of G1 and in G2, indicating possible long-distance transmission events and gene flow.

**Figure 4:**
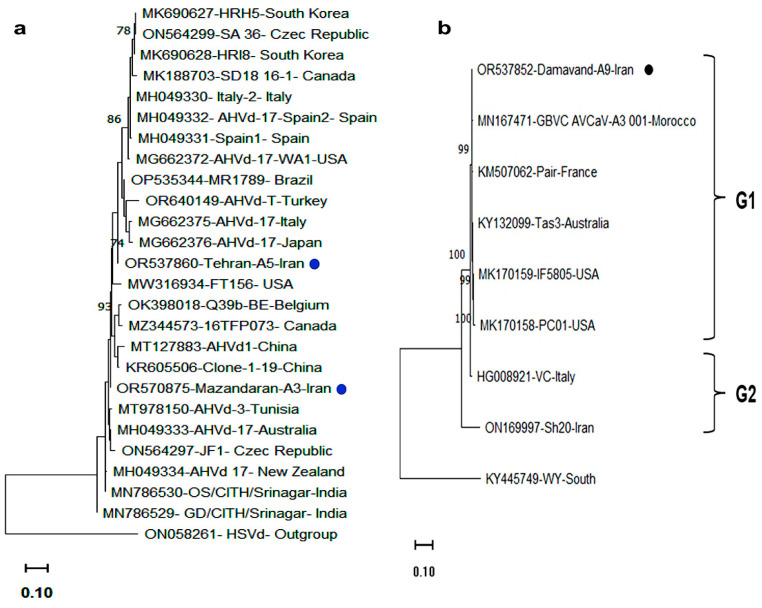
Phylogenetic trees based on the full-length genomic sequences of (**a**) AHVd isolates from apple trees in Mazandaran and Tehran provinces, and (**b**) AVCaV isolates from apple trees in Tehran province, Iran, together with homologous sequences retrieved from the NCBI database. Isolates are labeled by accession number/isolate name/country. Trees were constructed in MEGA 11.0 using the maximum likelihood (ML) method with the Kimura two-parameter model and 1000 bootstrap replicates. Only bootstrap values above 70% are shown. Scale bars represent the number of nucleotide substitutions per site. Iranian isolates detected in this research are marked with black dots. *Hop stunt viroid* (HSVd; ON058261) and *Cherry virus A* (KY445749) were used as outgroup sequences for the AHVd and AVCaV phylogenies, respectively. See Table 1 and Table 4 for accession numbers.

The whole genome phylogenetic analysis of AVCaV, including Damavand-A5 and other full-length sequences from GenBank, revealed two distinct groups (Figure 4). Group I included most isolates regardless of host species, while the Iranian almond isolate Sh20 and the Italian apricot isolate VC formed Group II. Iranian sequences were distributed across both groups.

The complete genomes of AHVd and HSVd were amplified to confirm their presence. Amplicons of 435 bp (AHVd) and 300 bp (HSVd) were obtained. The AHVd isolate Mazandaran-A3 (OR570875) showed 92% nt identity to the Chinese apple isolate 1–9 (KR605506), similar to the Tehran-A5 isolate (OR537860) recovered by the NGS. Iranian AHVd variants were most divergent from the Italian isolate VC (MG662375). HSVd isolate Mazandaran-A9 (OR570874) showed 97.6% identity to Iranian isolates HM6 and IR-Gala (OQ124154, MN695313), but only 93.3% identity to the NGS-recovered Mazandaran-A7 isolate (OR537861), suggesting intra-regional diversity.

Phylogenetic analysis of AHVd based on 435 nt sequences from 23 global isolates revealed two major clades (Figure 4). Iranian AHVd isolates were placed in separate clades, clustering with isolates from China, Japan, Turkey, and Italy. This represents the first report of AHVd in apple trees in different apple cultivars showing AMD symptoms in central and northern regions of Iran.

## 4. Discussion

Next-generation Sequencing has become the method of choice for comprehensive virus identification in fruit trees, particularly for diseases of unclear etiology. In this study, a deep sequencing technique was applied for the first time to investigate the virome of AMD-affected apple trees in major cultivation regions of Iran. Seven viruses and two viroids were identified, with AGCaV, AVCaV, CCGaV, and ApMV being reported for the first time in apple trees in Iran. ACLSV, ASGV, ASPV, HSVd, and AHVd had been previously reported from apple in the country [17,18,19,20,21,25]. Among the findings, AVCaV was identified in apple, a non-Prunus species, for the first time worldwide, as it was previously only associated with stone fruits [26]. The Iranian AVCaV isolate clustered phylogenetically with global isolates irrespective of host but was distinct from the Iranian almond-infecting strain, indicating the presence of divergent AVCaV lineages within the country that may reflect host-specific adaptation. Furthermore, the relatively high detection rate of AVCaV (20.5%) supports its successful establishment in apple trees. Frequent recombination events among various members of the *Betaflexiviridae* family, including AVCaV within the genus *Prunevirus*, have been previously reported [27,28]. Moreover, several studies have suggested that reassortment and recombination within genetically diverse viral populations may enhance viral fitness and facilitate adaptation to new hosts [29]. Further research is needed to determine whether recombination plays a significant role in the evolution of AVCaV in newly identified hosts in Iran.

The CCGaV isolate showed higher sequence identity to apple-infecting isolates from China than to citrus-infecting strains, aligning with previous findings that suggest host-driven genomic differentiation [30]. Although CCGaV and AHVd have been associated with symptomatic infections in other hosts, no specific symptoms were observed in association with these pathogens in Iranian apple cultivars, likely due to the masking effect of co-infecting viruses.

Overall virus detection rates ranged from 47.6% for AGCaV to 4.7% for CCGaV, indicating a poor phytosanitary status in Iranian apple orchards. High infection rates of *Betaflexiviridae* members (AGCaV, ACLSV, AVCaV, ASGV) were consistent with previous reports [6,14]. These viruses often occur in latent or mixed infections, with symptom expression influenced by cultivar, strain, and environmental conditions [31]. ACLSV, AVCaV, HSVd, and AHVd were most prevalent in Alborz province, while AGCaV dominated in Mazandaran. This regional variation may reflect mixed infections causing synergistic effects or genetic differences among local apple cultivars. The high virus prevalence in northern provinces likely results from elevated inoculum pressure and efficient virus spread, despite the absence of known natural vectors [32]. Although seed transmission has been proposed, it is not considered the main dissemination route [32]. The co-cultivation of susceptible and tolerant cultivars, the presence of reservoir trees, and the lack of strict phytosanitary measures have likely contributed to the widespread incidence of viruses associated with Apple Mosaic Disease (AMD) in these regions.

High infection levels were recorded in both commercial (Red Delicious, Golden Delicious) and local (Shafi Abadi) cultivars, with ‘Gala’ appearing more tolerant. Contrary to previous reports [19], viruses and viroids were also detected in ‘Starking’, indicating its susceptibility under current field conditions.

Mixed infections were common (67%), with HSVd frequently co-occurring with other viruses, except CCGaV. Despite these frequent associations, no clear link could be established between the presence of individual or mixed infections and AMD symptoms, like prior studies [6,30,33].

The phylogenetic analyses confirmed the presence of divergent variants, likely resulting from multiple introductions or local diversification of viruses. ASGV isolates clustered with previously reported Iranian and non-East Asian isolates, consistent with the hypothesis that ASGV originated in East Asia and spread globally. Despite its host range expansion, ASGV shows relatively low sequence variability, reflecting long-term adaptation to apple.

Although AHVd was recently detected in Iran by RT-PCR [20], this study is the first to characterize the full-length genome and molecular features of Iranian AHVd isolates, revealing high genetic diversity. Phylogenetic analysis allocated the Iranian AHVd isolates in separate clades with isolates from diverse geographic origins, indicating extensive sequence variability regardless of the geographic origin. Furthermore, HSVd isolates showed high similarity to previously reported Iranian variants, supporting earlier evidence of grapevine-derived introductions [25,34].

## 5. Conclusions

This study provides the first comprehensive virome analysis of symptomatic apple trees in Iran using NGS. Several viruses and viroids, including AGCaV, AVCaV, CCGaV, and ApMV, were identified in apple for the first time in the country. Notably, the detection of AVCaV in apple suggests a host range expansion for this Prunevirus. The molecular and phylogenetic analyses of AGCaV, ACLSV, ASGV, and AVCaV revealed considerable genetic diversity among Iranian isolates, highlighting the presence of divergent strains. These findings enhance our understanding of the molecular epidemiology of apple-infecting viruses in Iran and provide essential data to support the development of effective virus indexing programs and the propagation of virus-free planting material. This work contributes to the growing body of genomic data required for improved management of viral diseases in apple cultivation in Iran.

## Figures and Tables

**Table 1:** The genome size, organization, sequence similarity, and accession numbers of viruses identified from mosaic disease-affected apple leaf samples by next-generation sequencing. ^a^ AGCaV, apple green crinkle-associated virus; ASPV, apple stem pitting virus; ACLSV, apple chlorotic leaf spot virus; CCGaV, citrus concave gum-associated virus; ApMV, apple mosaic virus; ASGV, apple stem grooving virus; AVCaV, apricot clearing-associated virus; AHVd, apple hammerhead viroid; HSVd, hop stunt viroid. ^b^ RdRp, RNA-dependent RNA polymerase; MP, movement protein; CP, coat protein; TGB, triple gene block.

Virus Name ^a^	Genome Segment (RNA)	Genome Size (kb)	Sequence Similarity (%)	Accession No. Received	UTR-5′	ORF1 ^b^	ORF2	ORF3	ORF4	ORF5	UTR-3′	Reference Accession
ACLSV	1	7555	88	OR537850	1–151	152–5809 (Replicase)	5718–7100 (MP)	6784–7365 (CP)			7366–7550	M58152
AGCaV	1	9266	90	OR537851	1–60	61–6612 (Replicase)	6697–7350 (TGB1)	7352–7714 (TGB2)	7623–7835 (TGB3)	7924–9135 (CP)	9136–9266	HE963831
AVCaV	1	8358	98	OR537852	1–78	79–6144 (Replicase)	6144–7526 (MP)	7057–7722 (CP)	7790–8209 (Nucleic acid binding)		8209–8358	KM507062
ASPV	1	9258	86	OR537853	1–55	56–6603 (Replicase)	6702–7373 (TGB1)	7375–7737 (TGB2)	7646–7873 (TGB3)	7946–9136 (CP)	9137–9258	MZ148076
ASGV	1	6486	98	OR537854	1–28	29–6346 (Replicae and CP)	4780–5742 (MP)				6354–6486	MZ126527
CCGaV	1	6658	99	OR537855	1–70	71–6625 (RdRp)					6625–6658	MZ926713
	2	2706	99	OR537856	1–52	53–1276 (MP)	1580–2632 (NP)				2632–2706	MZ926714
ApMV	1	3440	89	OR537857	1–62	63–3206 (RdRp)					3207–3440	HE574162
	2	2979	90	OR537858	1–79	80–2707 (RdRp)					2708–2979	HE574163
	3	2056	90	OR537859	1–168	169–1029 (MP)	1126–1794 (CP)				1795–2056	HE574164
AHVd	1	435	92	OR537860	-	-	-	-	-	-	-	KR605506
HSVd	1	300	98	OR537861	-	-	-	-	-	-	-	MF576422

**Table 2:** Infection rates of ACLSV, ASPV, ASGV, AGCaV, AVCaV, ApMV, CCGaV, AHVd, and HSVd in apple samples collected from orchards in different geographical regions of Iran. ^a^ Data obtained by RT-PCR for tested viruses. ^b^ Apple samples infected with two or more virus and viroid. The highest incidence rate of detected viruses in each surveyed province is shown in bold.

Province	Tested Samples ^a^ (*n*)	AGCaV (*n*/%)	ACLS (*n*/%)	AVCaV (*n*/%)	ASGV (*n*/%)	ASPV (*n*/%)	CCGaV (*n*/%)	ApMV (*n*/%)	HSVd (*n*/%)	AHVd (*n*/%)	Mixed Infections ^b^ (*n*/%)
**Mazandaran**	56	39/69.6	32/57.1	10/17.8	10/17.8	11/19.6	4/7.1	3/5.3	18/32.1	11/19.6	30/53.5
**Semnan**	52	26/50.0	11/21.1	7/13.4	12/23.0	8/15.3	3/5.7	1/1.9	8/15.3	1/1.9	25/48.0
**Alborz**	32	9/28.1	21/65.6	15/46.8	4/12.5	3/9.3	1/3.1	2/6.2	13/40.6	8/25.0	23/72.0
**Tehran**	30	7/23.3	14/46.6	3/10.0	3/10.0	2/6.6	0/0.0	0/0.0	8/26.6	6/20.0	15/50.0
**Total**	170	81	78	35	29	24	8	6	47	26	114
**Mean %**	—	47.6	45.8	20.5	17.0	14.1	4.7	3.5	27.6	15.2	67.0

**Table 3:** Virus and symptoms associated with the mosaic-affected leaf samples in different cultivars of apples throughout surveyed regions. ND: Not detected; D: deformation; CL: chlorotic leaf spot; NL: necrotic leaf spot; Vc: vein clearing.

**Origin of the Samples**	**Cultivar**	**AGCaV**		**ACLSV**		**ASGV**		**ASPV**		**AVCaV**	
		**No. Infected Samples**	**Symptoms**	**No. Infected Samples**	**Symptoms**	**No. Infected Samples**	**Symptoms**	**No. Infected Samples**	**Symptoms**	**No. Infected Samples**	**Symptoms**
**Mazandaran**	**Gala**	3	Cl, Vc	2	NL, D	1	Vc, Cl	3	D, Vc	ND	-
	**Golden Delicious**	11	D, Vc	9	NL, D	3	Vc, Cl	ND	-	2	Vc
	**Red Delicious**	13	D, Cl	8	CL, D	4	Vc, Cl	1	D, Cl	5	Vc, D
	**Shafi Abadi**	7	D, NL	12	D, NL	3	Vc, Cl	5	D, NL	3	Vc
	**Starking**	2	CL, Vc	1	Vc	ND	-	ND	-	ND	-
	**Granny Smith**	3	CL, Vc	ND	-	ND	-	2	D, Vc	ND	-
**Semnan**	**Gala**	1	D, Vc	ND	-	ND	-	3	D, Vc	ND	-
	**Golden Delicious**	5	M, Vc	1	CL	ND	-	1	CL, D, NL	2	Vc
	**Red Delicious**	6	D, NL	3	D, CL	2	Vc, NL	2	D, Vc	4	Vc, D
	**Shafi Abadi**	10	D, NL	6	D, Vc	7	Vc, Cl	1	-	1	Vc
	**Starking**	2	NL, D	ND	-	2	Vc, Cl	1	D, Vc	ND	-
	**Granny Smith**	2	CL	1	CL	1	Vc, Cl	ND	CL, D, NL	ND	-
**Alborz**	**Gala**	ND	-	3	NL, D	ND	-	ND	-	ND	-
	**Golden Delicious**	2	CL, Vc	4	D, Vc	1	Vc, Cl	ND	-	6	Vc
	**Red Delicious**	1	D, Cl	5	CL, D, NL	ND	-	1	D, CL	6	Vc, D
	**Shafi Abadi**	3	D, NL	7	D, Vc	3	Vc, Cl	2	D, CL	3	Vc
	**Starking**	2	D, Vc	ND	-	ND	-	ND	-	ND	-
	**Granny Smith**	1	Vc	2	CL, NL	ND	-	ND	-	ND	-
**Tehran**	**Gala**	ND	-	ND	-	ND	-	ND	-	ND	-
	**Golden Delicious**	1	D, Vc	2	D, NL	1	Vc, Cl	1	Vc, D	ND	Vc
	**Red Delicious**	2	D, CL	4	D, Vc	ND	-	1	Vc, Cl	1	Vc
	**Shafi Abadi**	4	D, NL	6	CL, NL, Vc	2	Vc, Cl	ND	-	2	Vc, D
	**Starking**	ND	-	ND	-	ND	-	ND	-	ND	-
	**Granny Smith**	ND	-	2	Vc, CL	ND	-	ND	-	ND	-
**Origin** **Of the samples**	**Cultivar**	**ApMV**		**CCGaV**		**AHVd**		**HSVd**			
		**No. Infected samples**	**Symptoms**	**No. Infected samples**	**Symptoms**	**No. Infected samples**	**Symptoms**	**No. Infected samples**	**Symptoms**		
**Mazandaran**	**Gala**	ND	-	ND	-	3	CL	2	NL		
	**Golden Delicious**	ND	-	1	D	1	D, CL	5	D, CL		
	**Red Delicious**	1	CL, NL	2	D, Vc	2	NL	4	Vc, CL		
	**Shafi Abadi**	2	D, CL	1	D	3	D, CL	5	D, CL		
	**Starking**	ND	-	ND	-	1	D, CL	1	D		
	**Granny Smith**	ND	-	ND	-	1	D, CL	1	D, CL		
**Semnan**	**Gala**	ND	-	ND	-	ND	-	ND	-		
	**Golden Delicious**	1	Vc, CL	3	D, NL	ND	-	3	D, NL		
	**Red Delicious**	ND	-	ND	-	ND	-	2	D, NL		
	**Shafi Abadi**	ND	-	ND	-	1	D, NL	1	CL, D		
	**Starking**	ND	-	ND	-	ND	-	1	CL		
	**Granny Smith**	ND	-	ND	-	ND	-	1	CL		
**Alborz**	**Gala**	ND	-	ND	-	2	D, Vc	ND	-		
	**Golden Delicious**	1	D, CL, NL	1	D, NL	1	D, CL	5	CL, Vc		
	**Red Delicious**	ND	-	ND	-	2	D, CL, NL	3	CL, D		
	**Shafi Abadi**	1	D, CL	ND	-	2	D	4	CL, D		
	**Starking**	ND	-	ND	-	ND	-	ND	-		
	**Granny Smith**	ND	-	ND	-	1	D	1	CL		
**Tehran**	**Gala**	ND	-	ND	-	ND	-	1	CL		
	**Golden Delicious**	ND	-	ND	-	1	CL, NL	1	CL		
	**Red Delicious**	ND	-	ND	-	ND	-	2	CL, D		
	**Shafi Abadi**	ND	-	ND	-	3	D, CL	3	CL, D, CL		
	**Starking**	ND	-	ND	-	ND	-	CL	NL		
	**Granny Smith**	ND	-	ND	-	2	D, NL	1	CL		

**Table 4:** Values from pairwise sequence comparisons based on BLAST analysis of the CP gene among ACLSV, ASGV, and AGCaV isolates from Iran and worldwide. ^a^ The CP sequence of Canadian isolate Aurora-1 (HE963831) used as a reference isolate for AGCaV. ^b^ The CP sequence of American isolate P863 (M58152) was used as a reference isolate for ACLSV. ^c^ The CP sequence of Canadian isolate 13TF138 (MZ126527) was used as a reference isolate for ASGV.

ASGV (aa)	ASGV (nt)	ACLSV (aa)	ACLSV (nt)	AGCaV (aa)	AGCaV (nt)	ASGV ^c^ Accession	ACLSV ^b^ Accession	AGCaV ^a^ Accession
100	99	92	84	85	84	OR537854 (Tehran-A4, Iran)	OR537850 (Damavand-A2, Iran)	OR537851 (Alborz-A5, Iran)
100	99	92	85	94	92	OR537865 (Damavand-A1, Iran)	OR537862 (Damavand-A, Iran)	OR570871 (AL22, Iran)
100	99	94	88	94	92	OR537866 (Alborz, Iran)	OR537863 (Semnan-A4, Iran)	OR570872 (MA10, Iran)
100	99	91	84	93	91	OR537867 (Mazandaran-A5, Iran)	OR537864 (Mazandaran-A5, Iran)	OR570873 (SEM19, Iran)
100	99	90	86	89	94	OQ263371 (K, Iran)	MK354028 (KS, Iran)	KT835289 (CYD, Italy)
100	99	90	87	85	82	OQ263370 (MN18, Iran)	MK354029 (MS, Iran)	MW842992 (Royal-Gala, Brazil)
100	99	92	84	100	100	OQ718412 (L57, Laos)	MK354022 (KSS, Iran)	HE963831 (Aurora-1, Canada)
100	99	91	86			MK354031 (KZ, Iran)	KM586376 (T12JA, Iran)	
100	99	91	84			MK354036 (X4, Iran)	KM586369 (T5Te, Iran)	
97	93	91	86			KR185346 (Cuiguan, China)	KM586363 (R2T6Na, Iran)	
100	99	89	87			MZ127820 (ASGV-MK, India)	MH534892 (DN11, China)	
100	99	88	84			OR640152 (ASGV_T, Turkey)	OR640150 (ACLSV-T, Turkey)	
95	90	94	87			LC184612 (Nagami, Japan)	AM494513 (Salooni, India)	
97	93	94	86			LC480456 (BH-Cnidium, South Korea)	KY579382 (16–133A-LT, Lithuania)	
100	99	95	87			MW322833 (ASGV/FT146, USA)	KX668485 (M075, Brazil)	
100	99	95	88			MW582790 (DSMZPV-0199, Germany)	KX224102 (ACLSV-Eth1, Ethiopia)	
100	99	95	88			AF438409 (UV01, Brazil)	ON641858 (LV-54, Latvia)	
98	90	94	86			LC645073 (ASGV-AK6, Japan)	LC006864 (PKO, South Korea)	
100	100	96	88			MZ126527 (13TF138, Canada)	MZ126517 (13TF180B, Canada)	
		94	87				LC533838 (RRACV2, Japan)	
		95	87				MK086027 (ACLSV-K2, Kazakhstan)	
		95	88				OQ731902 (DSMZ PV-1000, Germany)	
		92	90				ON641871 (UKR5–36, Ukraine)	
		93	86				HQ398253 (SK-92-pl, Slovakia)	
		84	80				EU223295 (TaTao5, USA)	
		100	100				M58152 (P863, USA)	

## Data Availability

Sequence data generated in this study are available from GenBank at https://www.ncbi.nlm.nih.gov/genbank/ accessed on 30 September 2024 with accession numbers provided in Table 3 and Table 4. All the data is available with the corresponding authors, and it will be made available on request.

## Supplementary Materials

The following supporting information can be downloaded at https://www.mdpi.com/article/10.3390/v17070979/s1. Supplementary Table S1: List of primers used in RT-PCR to detect apple viruses and viroids. Supplementary Table S2. Read statistics and percentage of virus and viroid reads obtained by next-generation sequencing Illumina NovaSeq 6000 via paired end sequencing method for two apple composite samples (A1 and A2).
