# Supplementary material for: Comprehensive Virome Profiling of Apple Mosaic Disease-Affected Trees in Iran Using RT-PCR and Next-Generation Sequencing
Source: Viruses. 2025 Jul 13;17(7):979. doi: 10.3390/v17070979 (PMC12298886; doi:10.3390/v17070979)
Supplement: Supplementary file 1 [file viruses-17-00979-s001.zip › viruses-3715875-supplementary.pdf]

Supplementary Table S1: List of primers used in RT-PCR to detect apple viruses and viroids

Viruses	Primer names	Primer sequences (5´-3´)	Amplicon size (bp)	Target genomic region	Reference
ACLSV	ACLSV-s<br>ACLSV-a	CATGGAAAGACAGGGGCAA<br>TGACTCTTTATACTCTTTCA	581	6784-7365 (CP)	This work
AGCaV	AGCaV-s<br>AGCaV-a	TCTTCARCCCCAATGGTTTCT<br>CCTGGGGTTCATATATTATGCT	630	7951-8581 (CP)	This work
ApMV	ApMV-s<br>ApMV-a	ATGGTCTGTAAGTACTGYGGTC<br>GTGGTAACTCACTCGTTATCAC	610	1126-1736 (CP)	This work
ASGV	ASGV-s<br>ASGV-a	ATGAGTTTGGAAGACGTGCTTC<br>CTCTCCGAACCYGCCTCGAAA	685	5640-6325 (CP)	This work
ASPV	ASPV-s<br>ASPV-a	ATGRCTTCCAATGGWTCYCAAC<br>GCAGTRCTTTCAACYCCAAAGA	1050	7956-9007 (CP)	This work
AVCaV	AVCaV-s<br>AVCaV-a	ATGTCTTTGAAAAATCAGAAA<br>ACAGCTCATAACTTGCAACCCT	665	7057-7722 (CP)	This work
CCGaV	CCGaV-CP-F<br>CCGaV-CP-R	CTTCAATTTAGATGTGAGTGGCA<br>GCTACTAGCCAGGATCAAGCA	999	1571-2571 (CP)	[15]
HSVd	HSVd-s<br>HSVd-a	GGCTCCTTTCTCAGGTAAG<br>CCGGGGCAACTCTTCTCAGAATCCA	300	1-300	[27]
AHVd	AHVd-13FPG<br>AHVd-12RPG	CCTTCCTGATGAGTCCGTTCCA<br>CTAATAGCCTCCGACCGTCAT	433	1-433	[28]
ASSVd	ASSVd-s<br>ASSVd-a	GGTAAACACC GTGCGGTTCC<br>GGGAAACACCTATTGTGTTTT	329	1-329	[29]

Supplementary Table S2. Read statistics and percentage of virus and viroid reads obtained by next-generation sequencing Illumina NovaSeq 6000 via paired end sequencing method for two apple composite samples (A1 and A2).

	Sample A1	Sample A2
**Total No. of Reads**	36,652,324	43,845,194
**Reads Mapped to Malus domestica**	31,309,559	40,819,590
**Reads Not Mapped to Malus domestica**	5,315,765	3,025,604
**Total No. of Viral Reads**	143563	225741
**Total No. of Viroidal Reads**	52943	105278
**Percentage of Viral Reads**	0.39	0.51
**Percentage of Viroidal Reads**	0.14	0.24
